# M2 vessel occlusion characteristics and outcome after endovascular therapy: A post-hoc pooled analysis of MR CLEAN MED, NO-IV and LATE

**DOI:** 10.1177/15910199251349012

**Published:** 2025-06-24

**Authors:** Sterre Dassen, Quirien Robbe, Bart Wagemans, Robrecht Knapen, Susan Olthuis, Linda Jacobi, Christiaan van der Leij, Urs Fischer, Elyas Ghariq, Nyika Kruyt, Wouter van der Steen, Natalie LeCouffe, Aad van der Lugt, Charles Majoie, Wim van Zwam, Adriaan van Es, Julie Staals

**Affiliations:** 1 118066 School for Cardiovascular Diseases (CARIM), Maastricht University Medical Center+, Maastricht, the Netherlands; 2 Department of Neurology, 199236 Maastricht University Medical Center+, Maastricht, the Netherlands; 3 Department of Radiology and Nuclear Medicine, 522567 Maastricht University Medical Center, Maastricht, the Netherlands; 4 168089 Mental Health and Neuroscience (MHeNs) Research Institute, University Maastricht, Maastricht, the Netherlands; 5 GROW, 568601 School for Oncology and Reproduction, 5211 Maastricht University, Maastricht, the Netherlands; 6 Department of Neurology, University Hospital Bern, University of Bern, Bern, Switzerland; 7 Department of Radiology, 2901 Haaglanden Medical Center, The Hague, the Netherlands; 8 University Neurovascular Centre (UNVC) Leiden-The Hague, Leiden, the Netherlands; 9 Department of Neurology, 4501 Leiden University Medical Center, Leiden, the Netherlands; 10 Department of Neurology, 6993 Erasmus MC University Medical Center, Rotterdam, the Netherlands; 11 Department of Neurology, 1209 Amsterdam UMC location University of Amsterdam, Amsterdam, the Netherlands; 12 Department of Radiology and Nuclear Medicine, 6993 Erasmus MC University Medical Center, Rotterdam, the Netherlands; 13 Department of Radiology and Nuclear Medicine, 26066 Amsterdam UMC, Amsterdam, the Netherlands; 14 Department of Radiology, 4501 Leiden University Medical Center, Leiden, the Netherlands

**Keywords:** Stroke, thrombectomy, middle cerebral artery

## Abstract

**Purpose:**

Endovascular treatment (EVT) of M2-segment occlusions in acute ischemic stroke patients is still under debate. The impact of different M2 vessel occlusion characteristics on the outcomes of EVT remains unclear. We evaluated the association between M2 occlusion characteristics and clinical and safety outcomes following EVT.

**Methods:**

This is a retrospective pooled post-hoc analysis of data from the MR CLEAN MED, MR CLEAN NO-IV and MR CLEAN LATE trials, including patients who underwent EVT for M2 occlusions. We classified M2 occlusions on CTA images by location (proximal/distal), vessel dominance, affected branch (superior/inferior) and hemisphere. The primary outcome was the 24-hour National Institutes of Health Stroke Scale (NIHSS) score. Secondary outcomes included ΔNIHSS, a 90-day modified Rankin Scale (mRS) score, EVT procedural characteristics and safety outcomes. We adjusted for relevant prognostic factors.

**Results:**

181 patients with endovascular-treated M2 occlusions were included. There were no significant differences in 24-hour NIHSS or ΔNIHSS between proximal and distal occlusions. Ordinal shift analysis of mRS showed similar outcomes for proximal and distal M2 occlusions (cOR 1.32, 95% CI 0.70–2.49). Vessel dominance, affected branch and hemisphere did not significantly influence the NIHSS, ΔNIHSS or 90-day mRS. More symptomatic intracranial haemorrhages were seen in EVT of inferior branch occlusion (9.1% versus 2.1%, *p* = 0.02).

**Conclusion:**

In patients with endovascular-treated M2 occlusions, our study suggests no significant differences in clinical outcomes based on occlusion location, vessel dominance, affected branch or hemisphere; however, confirmation from larger studies is required. Notably, the increased rate of symptomatic haemorrhage in EVT of inferior branch occlusions needs further exploration.

## Introduction

The initial Multicenter Randomized Clinical trial of Endovascular treatment of Acute ischemic stroke in the Netherlands (MR CLEAN) trial and subsequent studies have shown the efficacy and safety of endovascular therapy (EVT) in patients with an acute ischemic stroke due to a large vessel occlusion.^1–3^ With increasing experience in EVT and newly developed thrombectomy devices, more distally located occlusions may be recanalized as well. However, recent randomized controlled trials (RCTs) investigating EVT for distal medium vessel occlusions have found that EVT did not result in a significant reduction in disability or mortality compared to the best medical treatment alone.^4,5^ While some of the recent RCTs applied inclusion criteria based on vessel-specific characteristics, such as vessel dominance (one M2 branch with a larger arterial diameter than the other), other characteristics such as the location of the occlusion (proximal versus distal), affected branch (superior versus inferior branch) and affected hemisphere may influence clinical outcome in patients undergoing EVT for an M2 occlusion. Improving our understanding of these determinants may be relevant for treatment decisions.

In this study, we aim to assess and compare clinical outcomes, EVT procedural characteristics and safety outcomes in acute ischemic stroke patients with M2 occlusions that were treated with EVT, distinguishing between proximal and distal occlusions, dominant and co-/non-dominant branch occlusions, superior and inferior branch occlusions, and left and right affected hemisphere. We performed a pooled patient-level analysis based on the MR CLEAN MED, NO-IV and LATE trials.

## Methods

### Patient population

We used pooled data from the Multicenter Randomized Clinical trial of Endovascular treatment of Acute ischemic stroke in the Netherlands (MR CLEAN) MED trial, the MR CLEAN NO-IV trial and the MR CLEAN LATE trial that were performed between 2018 and 2022.^3,6,7^ The MR CLEAN-MED trial investigated the effect of periprocedural administration of heparin or aspirin, whereas the MR CLEAN-NO IV trial examined the effect of intravenous thrombolysis (IVT) prior to thrombectomy. The MR CLEAN LATE trial investigated the effect of EVT compared to best medical management in patients presenting between 6 and 24 hours after symptom onset or last known well. The MR CLEAN LATE trial selected patients based on collateral flow.

The trial populations included patients aged 18 years and older who had an acute ischemic stroke due to a proximal occlusion in the anterior circulation (ICA, ICA-T, M1 or M2), confirmed by computed tomography angiography (CTA). All patients had a neurological deficit of at least 2 on the National Institutes of Health Stroke Scale (NIHSS). The population included patients treated within the early window (<6 h, MRCLEAN MED and MRCLEAN NOIV) and in the late window (6–24 h, MR CLEAN LATE). Patients in the late window were selected on the presence of collateral flow in the middle cerebral artery (MCA) territory of the affected hemisphere. The specific exclusion criteria for each trial can be found in the respective protocols.^8–10^

**Figure 1.**
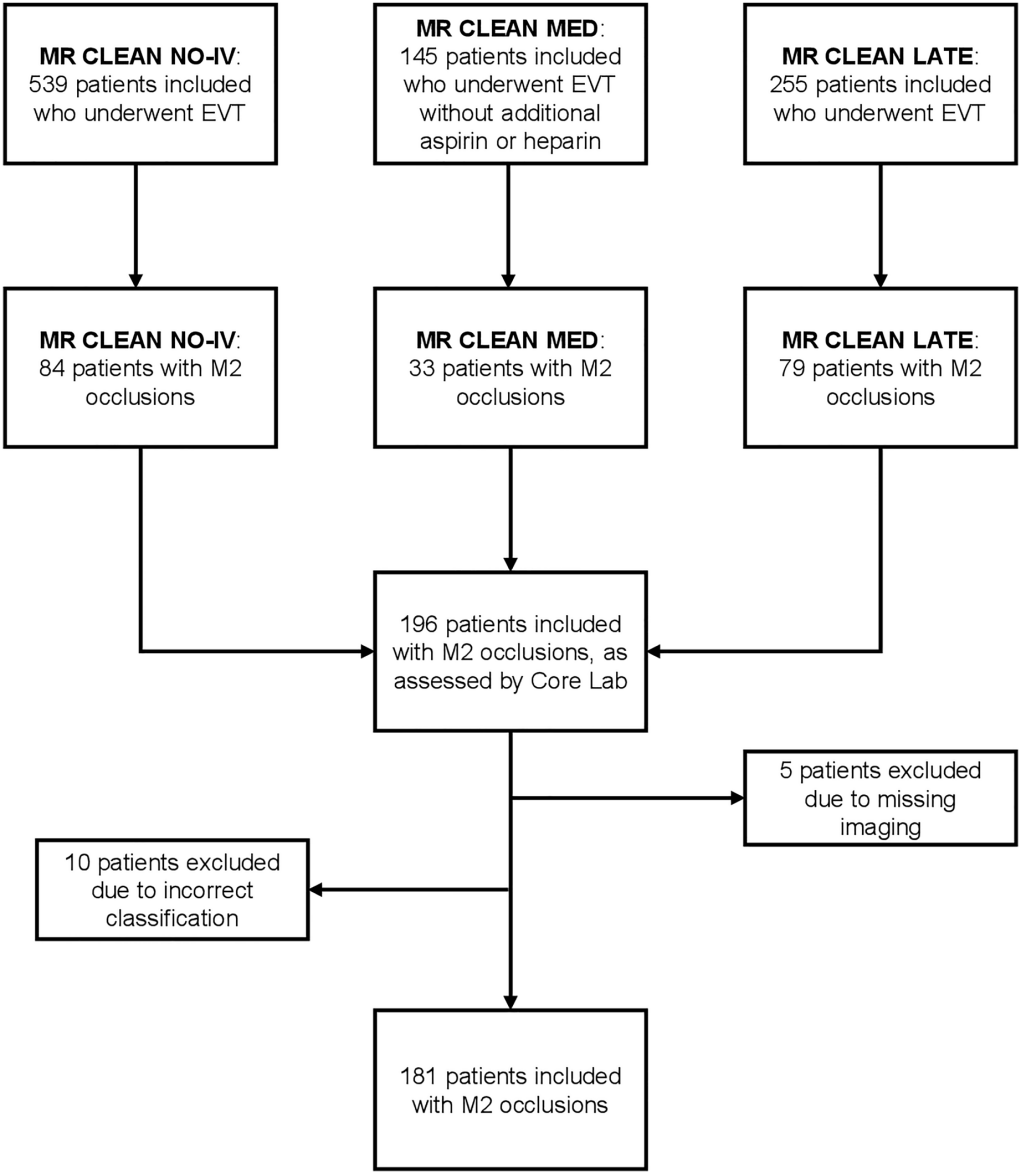
Flowchart of patient inclusion.

For this study, we included patients with an M2 occlusion identified on baseline CTA who underwent EVT. M2-segment occlusions were defined as any occlusion of the MCA distal to the first bifurcation, excluding the bifurcation with the anterior temporal branch of the MCA (Figure S1). From the MR CLEAN MED trial, we excluded the study arms with additional heparin or aspirin to avoid confounding effects of the medication on clinical outcomes. Additionally, we excluded patients for whom a pre-EVT CTA scan was unavailable. 181 patients met the final inclusion criteria (Figure 1).

### Imaging assessment

All baseline CTA images were reassessed by one of three blinded and experienced radiologists (AvE, BW, WvZ). The choice of imaging modality was left to the individual participating centres in the trials. The M2 segment was subdivided into 3 predefined parts, referred to as M2A, M2B and M2C (Figure S1). M2A was defined as the horizontal post-bifurcation segment of the M2. M2B was defined as the first half of the vertical segment of M2, starting at the curvature. Finally, M2C was defined as the second half of the vertical segment of M2. For the main analysis, M2A and M2B occlusions were regarded as proximal M2 occlusions. M2C occlusions were regarded as distal occlusions. The subdivision was made to address variations in the definition of distal occlusions in literature. In addition to the location of the occlusion, we assessed whether the occluded M2 vessel is dominant, co-dominant or non-dominant based on visual assessment of the arterial diameter. Finally, we assessed whether the occlusion was in the superior or inferior branch. We used the core laboratory assessment for the affected hemisphere, collateral score and post-EVT recanalization. The collateral score was assessed on CTA using the four-point score defined by Tan et al.^11,12^ Good collaterals were defined as collateral supply filling >50% of the occluded MCA territory. The digital subtraction angiography (DSA) images were assessed by the core laboratory for recanalization based on expanded treatment in cerebral infarction (eTICI) score. Successful recanalization was defined as eTICI ≥ 2B.

### Study parameters

The primary outcome variable was stroke severity, measured as NIHSS score at 24 h ranging from 0 (no deficit) to 42 (maximum deficit). As M2 occlusions typically result in smaller affected brain areas and less severe strokes compared to M1 occlusions,^13^ outcome differences among the subcategories of M2 occlusions were expected to be small. Consequently, NIHSS was chosen as the primary outcome, instead of the more commonly used modified Rankin Scale (mRS).^14^ The NIHSS is more sensitive to subtle neurological changes, with a minimal NIHSS score difference of 2 generally considered as clinically meaningful. In contrast, the mRS may group patients with minor deficits into similar categories, limiting its ability to detect small outcome differences in M2 occlusions. Furthermore, NIHSS scores have strong prognostic value for long-term functional outcomes after stroke.^15^

Secondary outcomes included a change in NIHSS, calculated as the difference between NIHSS at 24 h and NIHSS at baseline (ΔNIHSS), a shift in modified Rankin Scale (mRS) score ranging from 0 (no disability) to 6 (death) and dichotomized mRS score of 0–2 (functional independence) versus 3–6 (functional dependence) at 90 days. EVT procedural characteristics included successful recanalization rate, procedure duration defined as time from groin puncture to successful recanalization or last contrast bolus and number of attempts. Safety outcomes were the occurrence of procedural complications, including dissection, perforation and vasospasm, symptomatic intracranial haemorrhages (sICHs) according to the Heidelberg bleeding classification^16^ and mortality within 90 days.

### Missing data

The total percentage of missing data in this study was 3.7%. The percentage of missing data was 0.2% for clinical outcome variables, 4.8% for EVT procedure data, and 5.3% for safety outcome variables.

Multiple Imputation by Chained Equation (MICE) was used to handle missing data. The imputation model included relevant covariates and outcome variables. The number of imputations was based on the fraction of missing information, using the R-package *HowManyImputations* devised by Von Hippel.^17^

### Statistical analysis

Patient characteristics were analyzed using descriptive statistics, based on the crude dataset. Differences in baseline variables were assessed using Wilcoxon's rank sum test, Pearson's χ^2^ test or Fisher's exact test as appropriate.

Primary, secondary and safety outcomes were compared for proximal versus distal, dominant versus co-/non-dominant and superior versus inferior branch M2 occlusions as well as for left versus right affected hemispheres.

Associations between vessel occlusion characteristics and NIHSS at 24 h and ΔNIHSS were analyzed using linear regression. The regression model was adjusted based on potential confounders derived from literature, including age, NIHSS score at baseline, dichotomized collateral score and time between stroke onset or last-seen-well and groin puncture.

Secondary outcomes and safety outcomes were analyzed using ordinal logistic regression, binary logistic regression, Fisher's exact test or Pearson's χ^2^ test as appropriate. Odds ratios with 95% confidence intervals were reported. All regression models were adjusted for the same covariates, provided that the sample size was sufficient. When the sample size was insufficient, no adjustments were made. However, a sensitivity analysis including covariate adjustments is presented in Supplemental Table S10.

Finally, a sensitivity analysis was performed where M2A occlusions were considered proximal M2 occlusions and M2B and M2C occlusions were considered distal M2 occlusions, in order to account for variations in the definition of distal M2 occlusions.

All tests were two-tailed. Statistical significance was set at *p* *<* 0.05. Statistical analyses were conducted using RStudio (version 4.3.2). Due to the exploratory nature of this research, no multiplicity corrections were performed.^18^

## Results

A total of 196 patients with EVT-treated M2 occlusions were recruited from the trials. After excluding 5 patients due to missing pre-EVT CTA imaging and 10 patients due to incorrect initial classification by the core laboratory as M2 occlusion, 181 patients remained for analysis (Figure 1). The subject count included in analyses by trial were MR CLEAN NO-IV (n = 76), MR CLEAN MED (n = 31) and MR CLEAN LATE (n = 74) (Figure 1).

### Occlusion location

A total of 146 (81%) occlusions were classified as proximal M2 occlusions (59 M2A and 87 M2B) and 35 (19%) as distal M2 occlusions (M2C).

Baseline characteristics of patients grouped in proximal (M2A and M2B) or distal (M2C) occlusion are described in Table 1. Supplemental Table S1 provides baseline characteristics stratified by each occlusion location.

**Table 1.** Baseline characteristics and outcome of patients treated for M2 occlusions stratified by occlusion location.

	Proximal (n = 146)	Distal (n = 35)	*p-*value
Age	71 (13)	72 (11)	>0.9
Sex – Male	77 (53%)	23 (66%)	0.2
Medical History			
History of ischemic stroke	31 (21%)	3 (8.6%)	0.09
History of atrial fibrillation	31 (21%)	2 (5.7%)	0.03
History of diabetes mellitus	25 (17%)	10 (29%)	0.12
Pre-stroke mRS*			0.6
0 – No symptoms	91 (63%)	26 (74%)	
1 – Minor symptoms, no limitations	32 (22%)	6 (17%)	
2 – Slight disability, no help needed	16 (11%)	3 (8.6%)	
mRS ≥ 3	6 (4.1%)	0 (0%)	
Systolic blood pressure* [mmHg]	157 (27)	152 (20)	0.4
IVT administered	53 (36%)	14 (40%)	0.7
Baseline NIHSS*	8 (5–13)	7 (5–11)	0.15
ASPECTS	9 (9–10)	10 (9–10)	0.2
Collateral score^†^			>0.9
Poor collaterals (filling <50% of occluded area)	31 (22%)	7 (21%)	
Good collaterals (filling >50% of occluded area)	113 (78%)	26 (79%)	
Onset to groin puncture^‡^ [min]	456 (389)	360 (311)	0.3
Affected hemisphere – Left	80 (55%)	21 (60%)	0.6
Vessel dominance			<0.001
Dominant	106 (73%)	14 (40%)	
Co-/non-dominant	40 (27%)	21 (60%)	
Affected branch			>0.9
Inferior	80 (55%)	19 (54%)	
Superior	66 (45%)	16 (46%)	
**Outcome**			
NIHSS at 24h	5 (1–12)	5 (2–10)	0.9
Delta NIHSS*	−3 (−7 to 1)	−3 (−4 to −1)	0.6
mRS at 90 days			0.7
0 – No symptoms	13 (8.9%)	3 (8.6%)	
1 – Minor symptoms, no limitations	27 (18%)	6 (17%)	
2 – Slight disability, no help needed	36 (25%)	12 (34%)	
mRS ≥ 3	70 (48%)	14 (40%)	

Mean (sd); n (%); median (IQR).

NIHSS: National Institutes of Health Stroke Scale, mRS: modified Rankin Scale, IVT: intravenous thrombolysis, ASPECTS: Alberta Stroke Program Early CT Score.

* Data were missing for one patient.

† Data were missing for four patients.

‡ Data were missing for six patients.

Regression analysis indicated no association between occlusion location and 24-hour follow-up NIHSS or the ΔNIHSS (Table 2). Median 24-hour follow-up NIHSS was similar for proximal and distal occlusions, 5 (IQR: 1–12) versus 5 (IQR: 2–10), respectively (*p* = 0.9). Additionally, ΔNIHSS did not differ significantly between proximal and distal occlusions (*p* = 0.6), with both occlusions showing a median decrease of 3 points on NIHSS (IQR −7 to 1 and IQR −4 to −1 respectively) after EVT (Table 1).

**Table 2.** Effect of M2 vessel occlusion characteristics on 24-hour NIHSS and delta NIHSS.

	β	95% CI	aβ	95% CI
**24H NIHSS** *				
Distal occlusion^‡^	−1.47	[−4.84–1.90]	−0.04	[−3.09–3.02]
Co-/non-dominant vessel occlusion^§^	−0.99	[−3.82–1.82]	−0.26	[−2.85–2.33]
Superior branch affected^‖^	−1.09	[−3.77–1.59]	−0.82	[−3.21–1.56]
Right hemisphere affected^#^	−1.92	[−4.59–0.75]	−1.62	[−4.02–0.78]
**Delta NIHSS** ^†^				
Distal occlusion^‡^	0.21	[−2.89–3.32]	0.63	[−2.47–3.72]
Co-/non-dominant vessel occlusion^§^	0.62	[−1.98–3.22]	−0.05	[−2.69–2.60]
Superior branch affected^‖^	−0.67	[−3.14–1.79]	−0.73	[−3.17–1.71]
Right hemisphere affected^#^	−1.89	[−4.35–0.57]	−1.61	[−4.06–0.85]

NIHSS: National Institutes of Health Stroke Scale.

* Adjusted for age, baseline NIHSS, dichotomized collateral score and time from onset to groin puncture.

† Adjusted for age, dichotomized collateral score and time from onset to groin puncture.

‡ Reference: Proximal occlusion.

§ Reference: Dominant vessel occlusion.

‖ Reference: Inferior branch affected.

# Reference: Left hemisphere affected.

**Figure 2.**
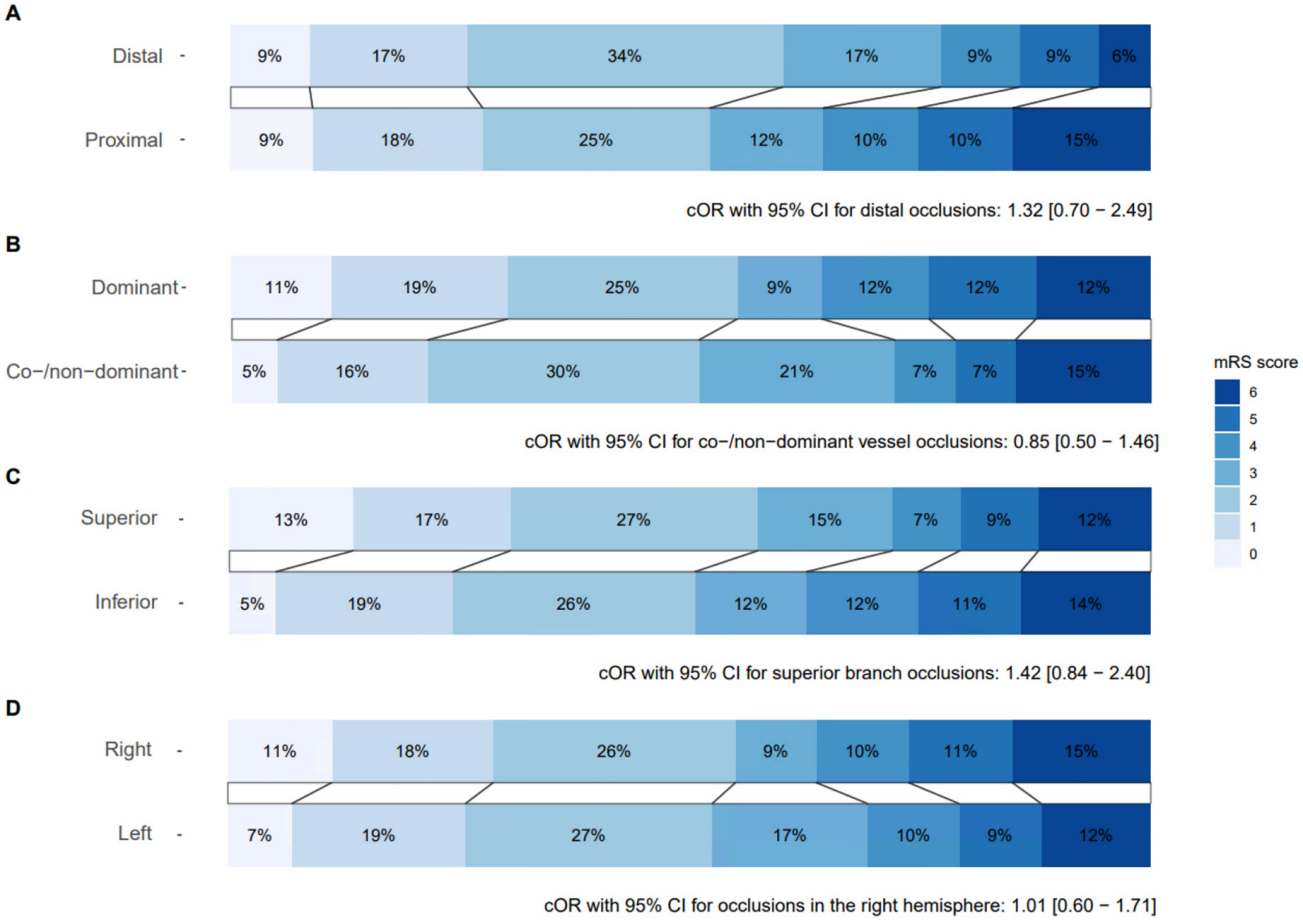
Distribution of the modified Rankin Scale (mRS) at 90 days with cOR and 95% CI for shift analysis stratified by (A) occlusion location − proximal (n = 146) and distal (n = 35), (B) vessel dominance − dominant (n = 120) and co-/non-dominant (n = 61), (C) affected branch − inferior branch (n = 99) and superior branch (n = 82), and (D) affected hemisphere − left hemisphere (n = 101) and right hemisphere (n = 80).

The distribution on the mRS scale at 90 days is shown in Figure 2(A). Functional independence (mRS 0–2) was achieved in 52% of patients with proximal occlusions and 60% of patients with distal occlusions. Shift analysis of the mRS score showed similar outcomes for proximal and distal M2 occlusions (cOR 1.32, 95% CI 0.70–2.49 for distal occlusions). No adjustments for covariates could be made due to the small sample size. Sensitivity analyses accounting for covariates showed comparable findings (Table S10).

Sensitivity analyses with an alternative definition of proximal (M2A) versus distal (M2B and M2C) occlusions yielded similar results (Figure S2, Tables S6, S7, S8 and S9).

### Vessel dominance

A total of 120 (66%) patients had a dominant vessel occlusion, while 61 (33%) patients had a co-/non-dominant vessel occlusion. Baseline patient characteristics stratified by vessel dominance can be found in Supplemental Table S2.

Dominance of the occluded vessel did not significantly influence the 24-hour follow-up NIHSS or ΔNIHSS (Table 2).

Functional independence was achieved in 51% of patients with co-/non-dominant vessel occlusions and in 55% of patients with dominant vessel occlusions (Figure 2(B)). Shift analysis of mRS score showed similar outcomes for dominant and co-/non-dominant vessel -occlusions (cOR 0.85, 95% CI 0.50–1.46 for co-/non-dominant vessel occlusions). No adjustments for covariates were made due to the small sample size. Sensitivity analyses accounting for covariates showed comparable findings (Table S10).

### Affected branch

When stratified by affected branch, 82 (45%) patients had an occlusion in the superior branch and 99 (55%) patients in the inferior branch. Baseline patient characteristics stratified by the affected branch are shown in Supplemental Table S3.

The affected branch did not significantly influence the 24-hour follow-up NIHSS or ΔNIHSS (Table 2).

Functional independence was achieved in 57% of patients with an occlusion in the superior branch and 51% of patients with an occlusion in the inferior branch (Figure 2(C)).

Ordinal shift analysis showed similar outcomes associated with an occlusion in the superior branch and an occlusion in the inferior branch (cOR 1.42, 95% CI 0.84–2.40 for an occlusion in the superior branch). Due to the small sample size, no adjustments for covariates were made. Sensitivity analyses accounting for covariates showed comparable findings (Table S10).

### Affected hemisphere

A total of 101 (56%) patients had an occlusion in the left hemisphere, while 80 (44%) patients had an occlusion in the right hemisphere. Baseline patient characteristics stratified by affected hemisphere are shown in Supplemental Table S4.

The affected hemisphere did not significantly influence the 24-hour follow-up NIHSS or ΔNIHSS (Table 2).

Functional independence was achieved in 52% of patients with an occlusion in the left hemisphere and 55% of patients with an occlusion in the right hemisphere (Figure 2(D)).

Shift analysis showed similar outcomes for patients with an occlusion in the right hemisphere and patients with an occlusion in the left hemisphere (cOR 1.01, 95% CI 0.60–1.71 for patients with an occlusion in the right hemisphere). No adjustments for covariates were made due to the small sample size. Sensitivity analyses accounting for covariates showed comparable findings (Table S10).

### EVT procedural characteristics

There were no significant differences in successful recanalization rates, procedure duration and total attempts between proximal versus distal occlusions, dominant versus co-/non-dominant branch occlusions, inferior versus superior branch occlusions and left versus right hemisphere occlusions (Table 3).

**Table 3.** EVT characteristics.

	Occlusion location		Vessel dominance		Affected branch		Affected hemisphere	
	Proximal (n = 146)	Distal (n = 35)	Dominant (n = 120)	Co-/non-dominant (n = 61)	Inferior (n = 99)	Superior (n = 82)	Left (n = 101)	Right (n = 80)
Duration of procedure* [min]	55 (32)	59 (30)	53 (28)	62 (39)	52 (28)	62 (36)	57 (34)	55 (30)
Total attempts^†^	2.1 (1.2)	2.1 (1.1)	2.1 (1.1)	2.1 (1.3)	2.0 (1.0)	2.2 (1.3)	2.0 (1.2)	2.2 (1.1)
Successful recanalization^‡^	96 (72%)	22 (69%)	81 (73%)	37 (67%)	68 (76%)	50 (66%)	70 (74%)	48 (67%)

Mean (sd); n (%).

* Data were missing for 10 patients.

† Data were missing for 1 patient.

‡ Data were missing for 15 patients.

### Safety outcomes

There were no significant differences in incidence rates of the safety outcomes between proximal and distal occlusions, dominant and co-/non-dominant vessel occlusions and left and right hemisphere occlusions (Table 4). Patients with an occlusion in the inferior branch had a significantly higher incidence rate of sICH compared to patients with an occlusion in the superior branch (9 patients [9.1%] versus 1 patient [2.1%], *p* = 0.02, Table 4). However, this did not reach significance in regression analysis (*p* = 0.051). Unadjusted and adjusted odds ratios with 95% CI for safety outcomes are shown in Supplemental Table S5. No adjusted odds ratio was reported for sICH due to the small sample size.

**Table 4.** Incidence of safety outcomes.

	Occlusion location			Vessel dominance			Affected branch			Affected hemisphere		
	Proximal (n = 146)	Distal (n = 35)	*p*-value*	Dominant (n = 120)	Co-/non-dominant (n = 61)	*p*-value*	Inferior (n = 99)	Superior (n = 82)	*p*-value*	Left (n = 101)	Right (n = 80)	*p*-value*
Procedural complication^†^	14 (11%)	2 (6.3%)	0.7	9 (8.3%)	7 (12%)	0.4	7 (7.8%)	9 (12%)	0.4	9 (10%)	7 (9.3%)	0.9
sICH^‡^	7 (4.8%)	3 (8.6%)	0.4	2 (3.3%)	8 (6.7%)	0.5	9 (9.1%)	1 (1.2%)	0.02	6 (5.9%)	4 (5.0%)	0.4
Mortality at 90 days	22 (15%)	2 (5.7%)	0.2	15 (13%)	9 (15%)	0.7	14 (14%)	10 (12%)	0.7	12 (12%)	12 (15%)	0.5

n (%).

sICH: symptomatic intracranial haemorrhage.

* Fisher's exact test; Pearson's Chi-squared test.

† Data were missing for 16 patients.

‡ Data were missing for 13 patients.

## Discussion

This study compared outcomes after EVT for patients with M2-segment occlusions further classifying them as proximal versus distal, dominant versus co-/non-dominant branch, superior versus inferior branch and left versus right affected hemisphere. We found no significant differences in clinical outcomes at 24 hours and 90 days and EVT procedural characteristics between these different subcategories. EVT in inferior branch occlusions showed a higher rate of sICH compared to superior branch occlusions, while other safety outcomes were similar across the subcategories.

Recent RCTs found no significant benefit of EVT over the best medical treatment for medium or distal vessel occlusions,^4,5^ although in the older EVT trials, a treatment effect has been observed in patients with proximal M2 occlusions.^13^ Our study, which included EVT-treated patients only, found no difference in clinical outcome between proximal and distal M2 occlusions. This may be explained by the larger affected brain area for proximal occlusions, leading to worse outcomes in the control group, while distal occlusions affect a smaller brain area and tend to have a more favourable natural outcome. As a result, the treatment effect appears more pronounced in proximal occlusions due to the greater disparity between control and treatment groups.

Despite a smaller calibre and more tortuous anatomy, successful recanalization in distal occlusions was achieved at a similar rate as in proximal occlusions, with a similar procedural duration and number of attempts in our study. This might reflect the growing experience of neuro-interventionists and the skilful selection of M2 occlusions assessed to be technically feasible. Recanalization rates reported in the literature range from 59%^13^ to over 90%,^19^ with the variance partially attributable to different technical approaches. The first study primarily employed stent retrievers, while the second utilized an aspiration distal access catheter. More research is needed to determine the effects of the different technical approaches in distal occlusions.

Furthermore, we found no significant differences in safety outcomes between proximal and distal occlusions, consistent with a previous study showing similar safety for EVT in both types of M2 occlusions.^20^ Although we used a different definition for proximal and distal occlusions, our sensitivity analyses using Weiss et al.'s definition confirmed our original findings that EVT is safe in both proximal and distal M2 occlusions. However, it should be noted that our sample size is likely too small to show a significant difference in a low incidence rate.

### Vessel dominance

Vessel dominance is used as a criterion for inclusion in several clinical trials investigating the clinical benefit of EVT in patients with an M2 occlusion.^21,22^ A previous prospective, observational cohort study showed that the clinical outcome for EVT in dominant M2 occlusions is similar to M1 occlusions,^23^ and the trials thus focus on co-/non-dominant M2 occlusions. We found no differences in clinical and safety outcomes between treated dominant and co-/non-dominant vessel occlusions, which is consistent with previous research^23^ and supports the rationale of conducting trials in co-/non-dominant vessel occlusions.

### Affected branch

When comparing EVT in superior and inferior branch occlusions, we found no differences in clinical outcomes. However, another study reported significantly higher functional independence rates for inferior division occlusions compared to superior division occlusions.^24^ Better outcomes may have been negated in our study by the significantly higher rates of sICH for EVT in the inferior branch. This did however not reach significance in unadjusted binary regression analysis, which may be due to the small sample size and the inherent conservativeness of binary regression. We have no clear explanation for the higher rate of sICH for EVT in the inferior branch. The inferior branch may technically be more challenging to treat, although, with the finding of similar recanalization rates and other complication rates, similar to that in the literature,^24^ inherent differences in the treatment of inferior and superior branches are not likely. Another hypothesis could be that ICH in the area of the inferior branch is more often symptomatic.

### Affected hemisphere

We found no differences in outcomes between EVT in right hemisphere stroke and left hemisphere stroke, similar to previous literature.^25^ Regression analysis showed lower NIHSS for right hemisphere strokes, although not significant. This may be attributed to a bias in the NIHSS, which assigns higher scores for left hemisphere strokes.^26,27^

### Strengths and limitations

A strength of this study is that it pooled prospectively collected data from three large multi-centre randomized controlled trials with assessment and scoring of the M2 subcategories by experienced neuroradiologists blinded to outcome variables and with limited missing data.

There are also a few limitations to acknowledge. A key limitation of our study is the relatively small sample size which restricted adjustment for covariates in regression analyses and may have prevented findings from reaching statistical significance due to insufficient power. As such, non-significant findings should be interpreted cautiously, and larger studies are needed to confirm our findings. Furthermore, this study lacks a non-EVT control group. Therefore, the treatment effect of EVT on proximal versus distal M2 occlusions could not be determined. Moreover, the number of distal M2 occlusions in this study was limited. In the MR CLEAN MED, MR CLEAN NO-IV and MR CLEAN LATE trial, only proximal M2 occlusions were eligible for inclusion. However, due to the lack of consensus on the definition of proximal and distal occlusions, some distal occlusions were included. Additionally, we cannot exclude selection bias related to the decision to include and treat a patient in the trials, and especially for the distal occlusions, it is possible that easier-to-treat patients were more commonly included. Finally, the differences in outcome between proximal and distal M2 occlusions appear to be small and our findings should be interpreted with caution and validated in future studies with a larger sample size.

## Conclusion

Given the recent neutral results of the RCTs investigating EVT in distal and medium vessel occlusions, we categorized patients who were treated for M2 occlusions based on vessel characteristics to explore whether certain subgroups exhibit different clinical outcomes after EVT. However, we found no significant differences in outcomes across these vessel characteristics. Further research is required to identify other factors that may influence outcomes after EVT in patients with M2 occlusions. The increased rate of sICH in EVT for the inferior M2 branch needs further study.

## Supplemental Material

sj-docx-1-ine-10.1177_15910199251349012 - Supplemental material for M2 vessel occlusion characteristics and outcome after endovascular therapy: A post-hoc pooled analysis of MR CLEAN MED, NO-IV and LATE Supplemental material, sj-docx-1-ine-10.1177_15910199251349012 for M2 vessel occlusion characteristics and outcome after endovascular therapy: A post-hoc pooled analysis of MR CLEAN MED, NO-IV and LATE by Sterre Dassen, Quirien Robbe, Bart Wagemans, Robrecht Knapen, Susan Olthuis, Linda Jacobi, Christiaan van der Leij, Urs Fischer, Elyas Ghariq, Nyika Kruyt, Wouter van der Steen, Natalie LeCouffe, Aad van der Lugt, Charles Majoie, Wim van Zwam, Adriaan van Es and Julie Staals in Interventional Neuroradiology
