# Supplementary material for: M2 vessel occlusion characteristics and outcome after endovascular therapy: A post-hoc pooled analysis of MR CLEAN MED, NO-IV and LATE
Source: Interv Neuroradiol. 2025 Jun 24:15910199251349012. Online ahead of print. doi: 10.1177/15910199251349012 (PMC12187717; doi:10.1177/15910199251349012)
Supplement: sj-docx-1-ine-10.1177_15910199251349012 - Supplemental material for M2 vessel occlusion characteristics and outcome after endovascular therapy: A post-hoc pooled analysis of MR CLEAN MED, NO-IV and LATE [file sj-docx-1-ine-10.1177_15910199251349012.docx]

**Supplemental Material**

Tables S1-S9

Figure S1-S2

| Table S1: Baseline characteristics and outcome stratified by occlusion location | | | | |
| --- | --- | --- | --- | --- |
|  | M2A (n=59) | M2B (n=87) | M2C (n=35) | *P* value |
| Age | 70 (12) | 72 (13) | 72 (11) | 0.6 |
| Sex – Male | 32 (54%) | 45 (52%) | 23 (66%) | 0.4 |
| Medical History |  |  |  |  |
| History of ischemic stroke | 14 (24%) | 17 (20%) | 3 (8.6%) | 0.2 |
| History of atrial fibrillation | 12 (20%) | 19 (22%) | 2 (5.7%) | 0.1 |
| History of diabetes mellitus | 6 (10%) | 19 (22%) | 10 (29%) | 0.07 |
| Pre-stroke mRS* |  |  |  | 0.8 |
| 0 - No symptoms | 36 (62%) | 55 (63%) | 26 (74%) |  |
| 1 - Minor symptoms, no limitations | 11 (19%) | 21 (24%) | 6 (17%) |  |
| 2 - Slight disability, no help needed | 8 (14%) | 8 (9.2%) | 3 (8.6%) |  |
| mRS ≥ 3 | 3 (5.2%) | 3 (3.4%) | 0 (0%) |  |
| Systolic blood pressure* [mmHg] | 154 (26) | 159 (29) | 152 (20) | 0.4 |
| IVT administered | 27 (46%) | 26 (30%) | 14 (40%) | 0.14 |
| Baseline NIHSS* | 9 (6-14) | 8 (5-13) | 7 (5-11) | 0.2 |
| ASPECTS | 9 (9 - 10) | 9 (9 - 10) | 10 (9 - 10) | 0.5 |
| Collateral score^†^ |  |  |  | 0.5 |
| Poor collaterals (filling <50% of occluded area) | 15 (26%) | 16 (18%) | 7 (21%) |  |
| Good collaterals (filling >50% of occluded area) | 42 (74%) | 71 (82%) | 26 (79%) |  |
| Onset to groin puncture^‡^ [min] | 373 (323) | 510 (418) | 360 (311) | 0.042 |

| Table S1: Baseline characteristics and outcome stratified by occlusion location | | | | |
| --- | --- | --- | --- | --- |
|  | M2A (n=59) | M2B (n=87) | M2C (n=35) | P value |
| Affected hemisphere – left | 29 (49%) | 51 (59%) | 21 (60%) | 0.5 |
| Vessel dominance |  |  |  | <0.001 |
| Dominant | 51 (86%) | 55 (63%) | 14 (40%) |  |
| Co-/non-dominant | 8 (14%) | 32 (37%) | 21 (60%) |  |
| Affected branch |  |  |  | 0.7 |
| Inferior | 35 (59%) | 45 (52%) | 19 (54%) |  |
| Superior | 24 (41%) | 42 (48%) | 16 (46%) |  |
| Outcome |  |  |  |  |
| NIHSS at 24h | 4 (1-14) | 5 (2-11) | 5 (2-10) | 0.9 |
| Delta NIHSS* | -3 (-8-0) | -2 (-6-1) | -3 (-4- -1) | 0.6 |
| mRS at 90 days |  |  |  | 0.8 |
| 0 - No symptoms | 7 (12%) | 6 (6.9%) | 3 (8.6%) |  |
| 1 - Minor symptoms, no limitations | 10 (17%) | 17 (20%) | 6 (17%) |  |
| 2 - Slight disability, no help needed | 13 (22%) | 23 (26%) | 12 (34%) |  |
| mRS ≥ 3 | 29 (49%) | 41 (47%) | 14 (40%) |  |
| Mean (sd); n (%); Median (IQR).  NIHSS: National Institutes of Health Stroke Scale, mRS: modified Rankin Scale, IVT: intravenous thrombolysis, ASPECTS: Alberta Stroke Program Early CT Score  * Data was missing for one patient.  † Data was missing for four patients.  ‡ Data was missing for six patients. | | | | |

| Table S2: Baseline characteristics and outcome stratified by vessel dominance | | | |
| --- | --- | --- | --- |
|  | Dominant (n=120) | Co-/non-dominant (n=61) | *P* value |
| Age | 72 (12) | 69 (12) | 0.08 |
| Sex – Male | 63 (53%) | 37 (61%) | 0.3 |
| Medical History |  |  |  |
| History of ischemic stroke | 24 (20%) | 10 (16%) | 0.6 |
| History of atrial fibrillation | 22 (18%) | 11 (18%) | >0.9 |
| History of diabetes mellitus | 26 (22%) | 9 (15%) | 0.3 |
| Pre-stroke mRS* |  |  | 0.7 |
| 0 - No symptoms | 81 (68%) | 36 (60%) |  |
| 1 - Minor symptoms, no limitations | 23 (19%) | 15 (25%) |  |
| 2 - Slight disability, no help needed | 12 (10%) | 7 (12%) |  |
| mRS ≥ 3 | 4 (3.3%) | 2 (3.3%) |  |
| Systolic blood pressure* [mmHg] | 157 (27) | 154 (25) | 0.3 |
| IVT administered | 49 (41%) | 18 (30%) | 0.14 |
| Baseline NIHSS* | 9 (5-14) | 7 (5-11) | 0.11 |
| ASPECTS | 10 (9 - 10) | 9 (8 - 10) | 0.002 |
| Collateral score^†^ |  |  | 0.9 |
| Poor collaterals (filling <50% of occluded area) | 26 (22%) | 12 (21%) |  |
| Good collaterals (filling of >50% of occluded area) | 93 (78%) | 46 (79%) |  |
| Onset to groin puncture^‡^ [min] | 378 (351) | 560 (400) | <0.001 |
| Affected hemisphere - Left | 66 (55%) | 35 (57%) | 0.8 |
| Affected branch |  |  | <0.001 |
| Inferior | 77 (64%) | 22 (36%) |  |
| Superior | 43 (36%) | 39 (64%) |  |
| **Outcome** |  |  |  |
| NIHSS at 24h | 5 (1-13) | 5 (2-9) | >0.9 |
| Delta NIHSS* | -3 (-7-0) | -3 (-5-0) | 0.3 |
| mRS at 90 days |  |  | 0.5 |
| 0 - No symptoms | 13 (11%) | 3 (4.9%) |  |
| 1 - Minor symptoms, no limitations | 23 (19%) | 10 (16%) |  |
| 2 - Slight disability, no help needed | 30 (25%) | 18 (30%) |  |
| mRS ≥ 3 | 54 (45%) | 30 (49%) |  |
| Mean (sd); n (%); Median (IQR).  NIHSS: National Institutes of Health Stroke Scale, mRS: modified Rankin Scale, IVT: intravenous thrombolysis, ASPECTS: Alberta Stroke Program Early CT Score  ^*^ Data was missing for one patient.  ^†^ Data was missing for four patients.  ^‡^ Data was missing for six patients. | | | |

| Table S3: Baseline characteristics and outcome stratified by affected branch | | | |
| --- | --- | --- | --- |
|  | Inferior (n=99) | Superior (n=82) | *P* value |
| Age | 71 (13) | 71 (11) | 0.6 |
| Sex – Male | 55 (56%) | 45 (55%) | >0.9 |
| Medical History |  |  |  |
| History of ischemic stroke | 14 (14%) | 20 (24%) | 0.08 |
| History of atrial fibrillation | 18 (18%) | 15 (18%) | >0.9 |
| History of diabetes mellitus | 23 (23%) | 12 (15%) | 0.14 |
| Pre-stroke mRS* |  |  | 0.8 |
| 0 - No symptoms | 62 (63%) | 55 (68%) |  |
| 1 - Minor symptoms, no limitations | 21 (21%) | 17 (21%) |  |
| 2 - Slight disability, no help needed | 12 (12%) | 7 (8.6%) |  |
| mRS ≥ 3 | 4 (4.0%) | 2 (2.5%) |  |
| Systolic blood pressure*^*^ [mmHg] | 156 (27) | 156 (25) | >0.9 |
| IVT administered | 38 (38%) | 29 (35%) | 0.7 |
| Baseline NIHSS* | 9 (5-13) | 7 (5-13) | 0.5 |
| ASPECTS | 10 (9 - 10) | 9 (8 - 10) | 0.6 |
| Collateral score^†^ |  |  | >0.9 |
| Poor collaterals (filling <50% of occluded area) | 21 (21%) | 17 (22%) |  |
| Good collaterals (filling >50% of occluded area) | 77 (79%) | 62 (78%) |  |
| Onset to groin puncture^‡^ [min] | 428 (377) | 451 (378) | 0.7 |
| Affected hemisphere – Left | 58 (59%) | 43 (52%) | 0.4 |
| Vessel dominance |  |  | <0.001 |
| Dominant | 77 (78%) | 43 (52%) |  |
| Co-/non-dominant | 22 (22%) | 39 (48%) |  |
| **Outcome** |  |  |  |
| NIHSS at 24h | 5 (1-13) | 5 (1-11) | 0.4 |
| Delta NIHSS* | -2 (-6-1) | -3 (-5-0) | 0.7 |
| mRS at 90 days |  |  | 0.2 |
| 0 - No symptoms | 5 (5.1%) | 11 (13%) |  |
| 1 - Minor symptoms, no limitations | 19 (19%) | 14 (17%) |  |
| 2 - Slight disability, no help needed | 26 (26%) | 22 (27%) |  |
| mRS ≥ 3 | 49 (49%) | 35 (43%) |  |
| Mean (sd); n (%); Median (IQR).  NIHSS: National Institutes of Health Stroke Scale, mRS: modified Rankin Scale, IVT: intravenous thrombolysis, ASPECTS: Alberta Stroke Program Early CT Score  ^*^ Data was missing for one patient.  ^†^ Data was missing for four patients.  ^‡^ Data was missing for six patients. | | | |

| Table S4: Baseline characteristics and outcome stratified by affected hemisphere | | | |
| --- | --- | --- | --- |
|  | Left hemisphere (n=101) | Right hemisphere (n=80) | *P* value |
| Age | 72 (12) | 70 (13) | 0.2 |
| Sex – Male | 56 (55%) | 44 (55%) | >0.9 |
| Medical History |  |  |  |
| History of ischemic stroke | 17 (17%) | 17 (21%) | 0.4 |
| History of atrial fibrillation | 22 (22%) | 11 (14%) | 0.2 |
| History of diabetes mellitus | 22 (22%) | 13 (16%) | 0.3 |
| Pre-stroke mRS* |  |  | 0.8 |
| 0 - No symptoms | 66 (65%) | 51 (65%) |  |
| 1 - Minor symptoms, no limitations | 23 (23%) | 15 (19%) |  |
| 2 - Slight disability, no help needed | 9 (8.9%) | 10 (13%) |  |
| mRS ≥ 3 | 3 (3.0%) | 3 (3.8%) |  |
| Systolic blood pressure* [mmHg] | 158 (28) | 153 (23) | 0.2 |
| IVT administered | 36 (36%) | 31 (39%) | 0.7 |
| Baseline NIHSS* | 7 (5-13) | 9 (6-13) | 0.5 |
| ASPECTS | 10 (9 - 10) | 9 (8 - 10) | 0.4 |
| Collateral score^†^ |  |  | 0.5 |
| Bad collaterals (filling <50% of occluded area) | 19 (19%) | 19 (24%) |  |
| Good collaterals (filling of >50% of occluded area) | 79 (81%) | 60 (76%) |  |
| Onset to groin puncture^‡^ [min] | 460 (377) | 412 (377) | 0.11 |
| Vessel dominance |  |  | 0.8 |
| Dominant | 66 (65%) | 54 (68%) |  |
| Co-/non-dominant | 35 (35%) | 26 (33%) |  |
| Affected branch |  |  | 0.4 |
| Inferior | 58 (57%) | 41 (51%) |  |
| Superior | 43 (43%) | 39 (49%) |  |
| **Outcome** |  |  |  |
| NIHSS at 24h | 5 (1-13) | 5 (1-10) | 0.3 |
| Delta NIHSS* | -2 (-5-1) | -4 (-7-0) | 0.14 |
| mRS at 90 days |  |  | 0.8 |
| 0 - No symptoms | 7 (6.9%) | 9 (11%) |  |
| 1 - Minor symptoms, no limitations | 19 (19%) | 14 (18%) |  |
| 2 - Slight disability, no help needed | 27 (27%) | 21 (26%) |  |
| mRS ≥ 3 | 48 (48%) | 36 (45%) |  |
| Mean (sd); n (%); Median (IQR).  NIHSS: National Institutes of Health Stroke Scale, mRS: modified Rankin Scale, IVT: intravenous thrombolysis, ASPECTS: Alberta Stroke Program Early CT Score  ^*^ Data was missing for one patient.  ^†^ Data was missing for four patients.  ^‡^ Data was missing for six patients. | | | |

| Table S5: Unadjusted and adjusted Odds Ratios with 95% confidence interval for safety outcomes | | | | |
| --- | --- | --- | --- | --- |
|  | OR | 95% CI | aOR | 95% CI |
| **Procedural complications*** ^†^ | | | | |
| Distal occlusion^§^ | 0.59 | [0.13 – 2.62] | 0.61 | [0.13-2.70] |
| Co-/non-dominant vessel occlusion^‖^ | 1.29 | [0.45 - 3.75] | 1.35 | [0.46-3.95] |
| Superior branch affected^#^ | 1.55 | [0.57 – 4.18] | 1.56 | [0.57-4.24] |
| Right hemisphere affected^**^ | 0.82 | [0.29 - 2.29] | 0.82 | [0.30-2.30] |
| **sICH**^‡^ | | | | |
| Distal occlusion^§^ | 1.86 | [0.45 – 7.67] | - | - |
| Co-/non-dominant vessel occlusion^‖^ | 0.47 | [0.10 – 2.33] | - | - |
| Superior branch affected^#^ | 0.12 | [0.02– 1.01] | - | - |
| Right hemisphere affected^**^ | 0.83 | [0.22 – 3.09] | - | - |
| **Mortality at 90 days*** | | | | |
| Distal occlusion^§^ | 0.34 | [0.08 – 1.54] | 0.41 | [0.08 – 1.93] |
| Co-/non-dominant vessel occlusion^‖^ | 1.21 | [0.49 – 2.97] | 1.13 | [1.06 – 1.21] |
| Superior branch affected^#^ | 0.84 | [0.35 – 2.03] | 0.86 | [0.35 - 2.16] |
| Right hemisphere affected^**^ | 1.31 | [0.55 – 3.11] | 1.42 | [0.57 – 3.53] |
| NIHSS: National Institutes of Health Stroke Scale, sICH: Symptomatic intracranial hemorrhage  *Adjusted for baseline NIHSS.  † Data was missing for 16 patients  ‡ Data was missing for 13 patients.  § Reference: Proximal occlusion  ‖ Reference: Dominant vessel occlusion  # Reference: Inferior branch affected  ** Reference: Left hemisphere affected | | | | |

| Table S6: Baseline characteristics and outcome stratified by occlusion location, defining M2A occlusions as proximal and M2B and M2C occlusions as distal. | | | |
| --- | --- | --- | --- |
|  | Proximal (n=59) | Distal (n=122) | *P* value |
| Age | 70 (12) | 72 (12) | 0.4 |
| Sex – Male | 32 (54%) | 68 (56%) | 0.8 |
| Medical History |  |  |  |
| History of ischemic stroke | 14 (24%) | 20 (16%) | 0.2 |
| History of atrial fibrillation | 12 (20%) | 21 (17%) | 0.6 |
| History of diabetes mellitus | 6 (10%) | 29 (24%) | 0.03 |
| Pre-stroke mRS* |  |  | 0.5 |
| 0 - No symptoms | 36 (62%) | 81 (66%) |  |
| 1 - Minor symptoms, no limitations | 11 (19%) | 27 (22%) |  |
| 2 - Slight disability, no help needed | 8 (14%) | 11 (9.0%) |  |
| mRS ≥ 3 | 3 (5.2%) | 3 (2.5%) |  |
| Systolic blood pressure* [mmHg] | 154 (26) | 157 (27) | 0.5 |
| IVT administered | 27 (46%) | 40 (33%) | 0.09 |
| Baseline NIHSS* | 9 (6-14) | 7 (5-12) | 0.14 |
| ASPECTS | 9 (9- 10) | 10 (9 - 10) | 0.7 |
| Collateral score^†^ |  |  | 0.3 |
| Bad collaterals (filling <50% of occluded area) | 15 (26%) | 23 (19%) |  |
| Good collaterals (filling of >50% of occluded area) | 42 (74%) | 97 (81%) |  |
| Onset to groin puncture^‡^ [min] | 373 (323) | 469 (397) | 0.07 |
| Affected hemisphere- Left | 29 (49%) | 72 (59%) | 0.2 |
| Vessel dominance |  |  | <0.001 |
| Dominant | 51 (86%) | 69 (57%) |  |
| Co-/non-dominant | 8 (14%) | 53 (43%) |  |
| Affected branch |  |  | 0.4 |
| Inferior | 35 (59%) | 64 (52%) |  |
| Superior | 24 (41%) | 58 (48%) |  |
| **Outcome** |  |  |  |
| NIHSS at 24h | 4 (1-14) | 5 (2-11) | 0.7 |
| Delta NIHSS* | -3 (-8-0) | -3 (-5-1) | 0.3 |
| mRS at 90 days |  |  | 0.6 |
| 0 - No symptoms | 7 (12%) | 9 (7.4%) |  |
| 1 - Minor symptoms, no limitations | 10 (17%) | 23 (19%) |  |
| 2 - Slight disability, no help needed | 13 (22%) | 35 (29%) |  |
| mRS ≥ 3 | 29 (49%) | 55 (45%) |  |
| Mean (sd); n (%); Median (IQR).  NIHSS: National Institutes of Health Stroke Scale, mRS: modified Rankin Scale, IVT: intravenous thrombolysis, ASPECTS: Alberta Stroke Program Early CT Score  ^*^ Data was missing for one patient.  ^†^ Data was missing for four patients.  ^‡^ Data was missing for six patients. | | | |

| Table S7: Effect of M2 vessel occlusion characteristics on 24-hour NIHSS and delta NIHSS, with M2A occlusions defined proximal and M2B and M2C occlusions as distal. | | | | |
| --- | --- | --- | --- | --- |
|  | β | 95% CI | aβ | 95% CI |
| **24H NIHSS**^∗^ | | | | |
| Distal occlusion^‡^ | -0.14 | [-2.99 – 2.70] | 0.35 | [-2.22 – 2.92] |
| **Delta NIHSS**^†^ | | | | |
| Distal occlusion^‡^ | 1.06 | [-1.56 – 3.68] | 0.65 | [-1.97 – 3.27] |
| NIHSS: National Institutes of Health Stroke Scale  *Adjusted for age, baseline NIHSS, dichotomized collateral score and time from onset to groin puncture.  † Adjusted for age, dichotomized collateral score and time from onset to groin puncture.  ‡ Reference: Proximal occlusion | | | | |

| Table S8: EVT procedure data | | |
| --- | --- | --- |
|  | Proximal (n=85) | Distal (n=110) |
| Duration of procedure* [min] | 53 (29) | 58 (35) |
| Total attempts^†^ | 2.1 (1.10) | 2.1 (1.26) |
| Successful recanalization^‡^ | 36 (68%) | 82 (73%) |
| Mean (sd); n (%)  ^*^Data was missing for 10 patients.  ^†^ Data was missing for 1 patient.  ^‡^ Data was missing for 15 patients. | | |

| Table S9: Unadjusted and adjusted Odds Ratios with 95% confidence interval for safety outcomes | | | | |
| --- | --- | --- | --- | --- |
|  | OR | 95% CI | aOR | 95% CI |
| **Procedural complications^*‡^** | | | | |
| Distal occlusion^‖^ | 0.67 | [0.24 - 1.87] | 0.68 | [0.24 - 1.10] |
| **sICH^†§^** | | | | |
| Distal occlusion^‖^ | 1.14 | [0.28 – 4.60] | - | - |
| **Mortality at 90 days^*^** | | | | |
| Distal occlusion^‖^ | 1.53 | [0.57 – 4.11] | 1.83 | [0.65 – 5.13] |
| NIHSS: National Institutes of Health Stroke Scale  sICH: Symptomatic intracranial hemorrhage  ^*^Adjusted for baseline NIHSS.  ^†^ Adjusted for age, baseline NIHSS, dichotomized collateral score and time from onset to groin puncture.  ^‡^ Data was missing for 16 patients  ^§^ Data was missing for 13 patients.  ^‖^ Reference: Proximal occlusion | | | | |

| Table S10: Ordinal logistic regression results for mRS adjusted for covariates | | |
| --- | --- | --- |
|  | acOR* | 95%-CI |
| Distal occlusions^†^ | 1.09 | [0.57 – 2.08] |
| Co-/non-dominant vessel occlusion^‡^ | 0.67 | [0.38 – 1.17] |
| Superior branch affected^§^ | 1.41 | [0.83 – 2.40] |
| Right hemisphere affected^‖^ | 1.02 | [0.60 – 1.74] |
| *Adjusted for age, baseline NIHSS, dichotomized collateral score and time from onset to groin puncture.  ^†^ Reference: Proximal occlusion  ^‡^ Reference: Dominant vessel occlusion  ^§^Reference: Inferior branch affected  ^‖^Reference: Left hemisphere affected | | |

**
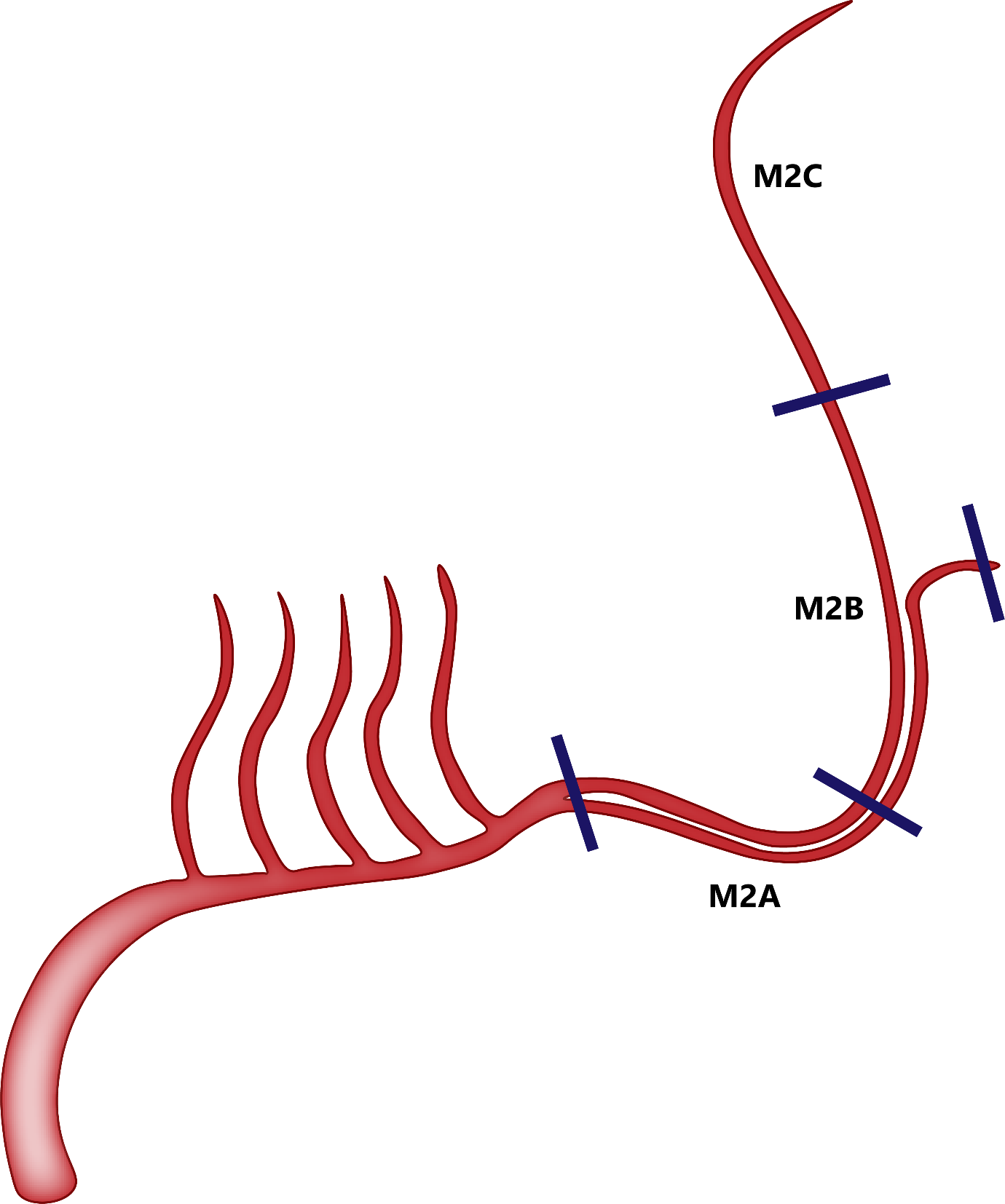
**

**Figure S1:** Graphical representation of M2-segment subdivision into M2A, M2B and M2C

**
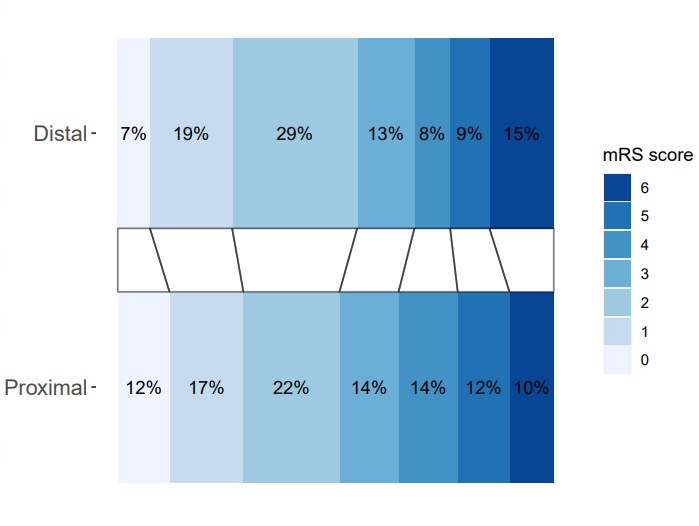
**

**Figure S2:** 90d mRS stratified by occlusion location, defining M2A occlusions as proximal (n=59) and M2B and M2C occlusions as distal (n=122)
